# "Visually impaired elderly patient ingests pill desiccant, leading to acute hypoxic respiratory failure requiring intubation"

**DOI:** 10.1186/s12877-017-0567-4

**Published:** 2017-07-28

**Authors:** Wendy Gerstein, Ziyang Liu

**Affiliations:** 1 Department of Internal Medicine, New Mexico VA Health Care Service, 1501 San Pedro Ave, SE, Albuquerque, NM 87108 USA; 2 Department of Internal Medicine Residency Program, University of New Mexico, 1 University of New Mexico, Albuquerque, NM 87131-0001 USA

**Keywords:** Adverse medication event, Patient safety, Functional status, Geriatric patients

## Abstract

**Background:**

A significant percentage of elderly patients suffer from both polypharmacy and visual impairment. This combination can increase the risk of an adverse event related to medication. This case highlights an unusual, but potentially deadly, medication adverse event.

**Case presentation:**

A 77-year-old male, visually impaired, ingested a pill desiccant, believing it was the ampicillin/sulbactam tablet he was prescribed for an infected diabetic foot ulcer. He presented to the emergency room with inability to swallow, and imaging revealed the pill desiccant lodged in his upper esophagus. He developed respiratory distress due to aspiration of secretions, necessitating intubation both to protect his airway and for an esophagogastroduodenoscopy (EGD). During EGD the desiccant was pushed into the stomach due to an inability to remove it without causing harm. Patient self-extubated the following day and per family and patient’s wishes was not re-intubated. The patient suffered no further complications directly related to the desiccant, but he died several days later from respiratory failure.

**Conclusions:**

This case highlights a concerning medication patient safety issue for visually impaired geriatric patients.

## Background

Worldwide, 285 million people are considered visually impaired based on the World Health Organization definition [1]. In the United States 6.5 million seniors over the age of 65 have a severe visual impairment, and this number is expected to double by 2030 [2]. In addition, 39% of patients over the age of 65 are on five or more prescribed medications [3]. Polypharmacy already has multiple inherent risks, and added is the concern of an inadvertent ingestion of a medication desiccant causing significant morbidity and mortality due to the cascade of events that can occur when a patient is hospitalized.

This following case report was structured using the CARE Statement Guidelines on reporting case reports [4].

## Case presentation

**Fig. 1:**
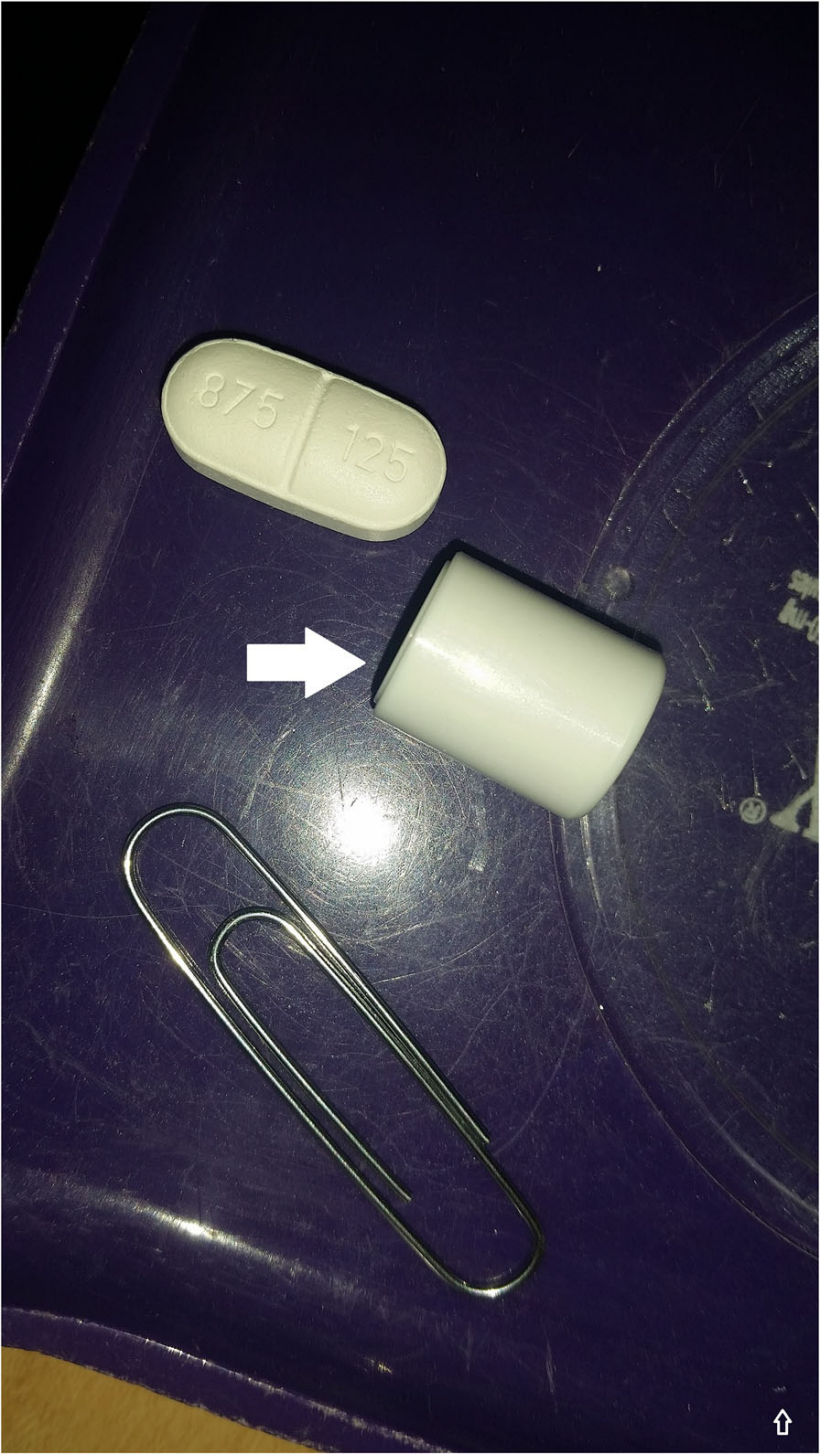
Pill and desiccant (filled arrow pointing to desiccant, paper clip for size reference)

The patient was a 77-year-old male with a history of diabetes complicated by severe diabetic retinopathy and vitreous hemorrhage who presented with an inability to swallow liquids or handle secretions after mistakenly taking the cylindrical desiccant instead of the amoxicillin/sulbactam tablet that he had been prescribed for an infected diabetic foot ulcer. The patient had decreased vision related to his retinopathy and was unable to tell the difference between the pill and the desiccant (Fig. 1). He had not been alerted to the desiccant presence when given the prescription.

**Fig. 2:**
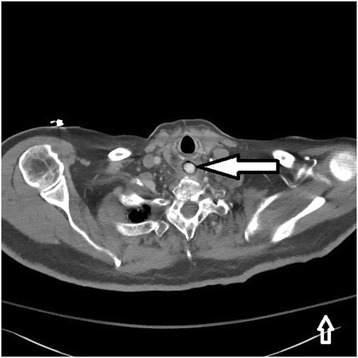
Coronal view, desiccant in upper esophagus (filled arrow pointing at desiccant)

**Fig. 3:**
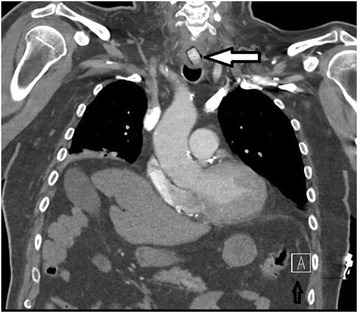
Sagittal view, desiccant in upper esophagus (filled arrow pointing at desiccant)

Shortly after presentation to the hospital the patient developed respiratory distress due to aspiration of his secretions and was intubated. Computed tomography of the thorax obtained prior to intubation confirmed a foreign object obstructing the esophagus (Figs. 2 and 3).

EGD was performed and the desiccant was pushed into the stomach due to inability to pull it upwards without causing harm. The following day patient self-extubated and recovered normal swallowing and bowel function but the offending desiccant was not recovered. Patient continued to have respiratory distress but family and patient declined re-intubation and patient died a few days later from hypoxic respiratory failure.

## Discussion and conclusions

Review of the literature reports four other cases of accidental desiccant ingestion presenting with different gastrointestinal complaints and complications [5–7]. Our case is the first known report of an accidental desiccant ingestion occurring due to impaired vision in an elderly patient causing acute respiratory failure, and highlights the importance of educating elderly patients with visual impairments the unique risks associated with taking medications as related to their decreased vision. A simple solution would be for pharmaceutical companies to ensure that the desiccant is a different color and shape than the medication, or as suggested by the authors of a prior case report, anchoring the desiccant to the bottom of the pill bottle. In addition, our patient, like almost 30% of U.S adults over 65 years of age, lived alone [8]. He was in frail health with recent history of hospitalizations, and had consistently refused in home services to assist with medication management. In similar cases, when patients are prescribed a new medication, pharmacy should specifically alert the patient to the presence of the desiccant. Our growing elderly population, with concurrent visual impairment and polypharmacy, necessitates that we address patient safety issues in a manner specifically tailored to the needs of our geriatric patients to optimize their health and outcomes.
